# Comparative Functional Genomics of Salt Stress in Related Model and Cultivated Plants Identifies and Overcomes Limitations to Translational Genomics

**DOI:** 10.1371/journal.pone.0017094

**Published:** 2011-02-14

**Authors:** Diego H. Sanchez, Fernando L. Pieckenstain, Jedrzey Szymanski, Alexander Erban, Mariusz Bromke, Matthew A. Hannah, Ute Kraemer, Joachim Kopka, Michael K. Udvardi

**Affiliations:** 1 Max Planck Institute for Molecular Plant Physiology (MPIMP), Potsdam-Golm, Germany; 2 Instituto Tecnológico de Chascomús (IIB-Intech), Chascomús, Argentina; 3 Department of Plant Physiology, Ruhr University Bochum, Bochum, Germany; 4 Samuel Roberts Noble Foundation, Ardmore, Oklahoma, United States of America; University of Toronto, Canada

## Abstract

One of the objectives of plant translational genomics is to use knowledge and genes discovered in model species to improve crops. However, the value of translational genomics to plant breeding, especially for complex traits like abiotic stress tolerance, remains uncertain. Using comparative genomics (ionomics, transcriptomics and metabolomics) we analyzed the responses to salinity of three model and three cultivated species of the legume genus *Lotus*. At physiological and ionomic levels, models responded to salinity in a similar way to crop species, and changes in the concentration of shoot Cl^−^ correlated well with tolerance. Metabolic changes were partially conserved, but divergence was observed amongst the genotypes. Transcriptome analysis showed that about 60% of expressed genes were responsive to salt treatment in one or more species, but less than 1% was responsive in all. Therefore, genotype-specific transcriptional and metabolic changes overshadowed conserved responses to salinity and represent an impediment to simple translational genomics. However, ‘triangulation’ from multiple genotypes enabled the identification of conserved and tolerant-specific responses that may provide durable tolerance across species.

## Introduction

Secondary salinization of soils caused by irrigation has become a major concern worldwide (www.fao.org/ag/agl/agll/spush). Salinity engenders both hyper-osmotic and hyper-ionic stresses, with plants facing dehydration, ion toxicity, nutritional deficiencies and oxidative stress [1]. Acclimation responses include ion exclusion and tissue tolerance, tight control of water homeostasis and osmotic adjustment, changes in growth and development, and a wide array of underlying biochemical and molecular changes [1]–[5]. Research on the molecular responses of plants to salinity has focused mostly on model species such as *Arabidopsis thaliana*, yet the value of model plants in identifying mechanisms that may confer stress tolerance to crops in the field remains to be seen [6]. Although it is known that salt tolerance is a quantitative trait determined by multiple and complex genetic interactions [7]–[9] and that plant responses to salinity involve changes in the expression of thousands of genes [2]–[3], [10], we know little about the extent of evolutionary conservation of molecular networks that determine salt tolerance. To understand better the nature of impediments that may stand in the way of translational genomics for salinity tolerance, we carried out a comparative functional genomic study between model and cultivated legumes of the genus *Lotus*.

Legumes are second only to grasses in their importance to agriculture, and provide a rich source of protein, oil, carbohydrate, minerals, and secondary compounds for human and animal nutrition [11]. The genomes of three legumes (*Lotus japonicus*, *Medicago truncatula* and *Glycine max*) have been sequenced, and it is envisioned that genomic discoveries in these species will be translated to crop improvement via breeding programs involving grain and forage legumes [12]. This approach seems reasonable given the high degree of synteny between legumes [13]. However, it remains to be seen whether orthologous genes that have significant effects on a complex, quantitative trait such as salinity tolerance in one species will have a similar effect in another.

In this study, we compared the physiological and molecular responses to salt stress of six *Lotus* species. Three of them, *L. japonicus*, *L. filicaulis*, and *L. burttii*, are in-breeding and have been used as models for legume genetics [14]–[20]. *L. japonicus* has been developed as a premier model, with genome sequence and numerous tools for genetics and genomics now available [10], [14]–[19]. *L. filicaulis* and *L. burttii* have been developed as crossing partners for genetic studies [20]. The other three species, *L. corniculatus*, *L. glaber* and *L. uliginosus*, are out-breeding and are used in world agriculture as forages. Although related, these species exhibit diversity in their ability to grow in low-fertility soils and under different environmental constraints [21]–[22]. Here, we present the results of comparative ionomic, transcriptomic and metabolomic analyses of *Lotus* genotypes that reveal conserved and divergent system responses to salinity within this genus. Our work has important implications for translational genomics approaches that aim to improve salinity tolerance and other complex traits in plants.

## Results

### Physiological and nutritional responses to salinity in *Lotus spp*

**Figure 1:**
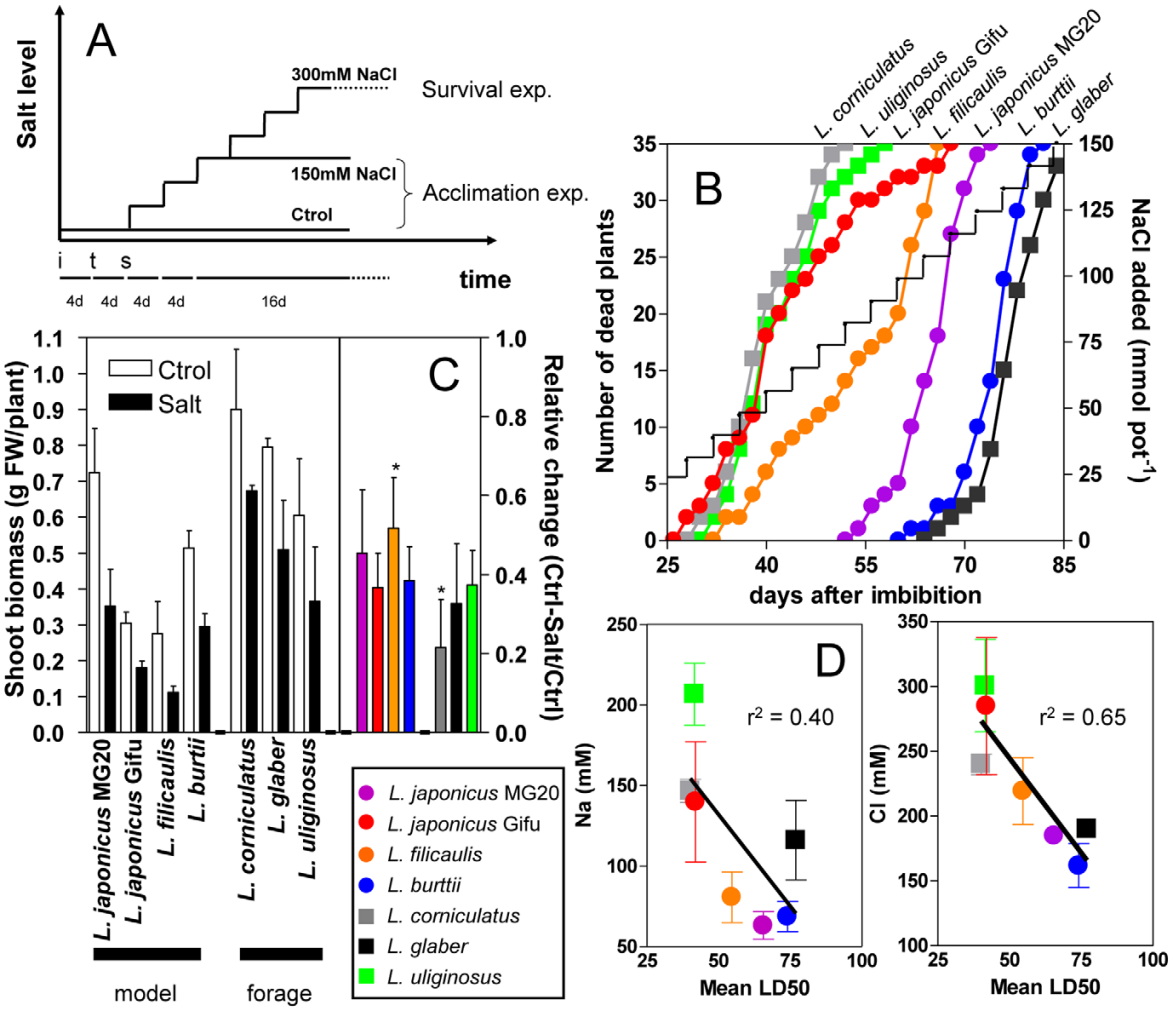
Experimental design and physiological assessment of salt tolerance and acclimation in *Lotus* species. (**A**) Experimental design for long-term survival (300 mM NaCl) and sub-lethal salt acclimation (150 mM NaCl) experiments. NaCl concentration in the nutrient solution was increased by 50 mM every four days (see Materials and methods). i =  seed imbibition, t =  transplanting, s =  start salinization, d =  days. (**B**) Representative experiment for survival of *Lotus* species under lethal NaCl-stress conditions. The step-wise increase in total NaCl added to each pot is estimated on the right axis. (**C**) Plant growth evaluated as final shoot biomass (left panel) and relative performance (right panel) under sub-lethal NaCl levels. Data represents the mean ± SD of 3 independent experiments, and the asterisk indicates a statistically-significant difference in stress-induced change in biomass between *L. corniculatus* and *L. filicaulis* (Student *t*-test, p<0.05). Model species are shown to the left. FW  =  fresh weight. (**D**) Linear correlation between Na^+^ or Cl^−^ content of salt-acclimated plants (shoots) and mean LD50 calculated from survival experiments (Table 1).

The relative salt tolerance of *Lotus* genotypes representing the six species described above, including two accessions of *L. japonicus* (MG20 and Gifu), was determined in two independent survival experiments in which plants were subjected to long-term step-wise increases in the level of NaCl up to 300 mM NaCl (Figure 1A and B). We defined the ‘lethal-dose fifty’ (LD50) as the number of days at which 50% of plants had died. The resulting ranking from most to least tolerant genotype was: *L. glaber > L. burttii* > *L. japonicus* var. MG20 > *L. filicaulis* > *L. japonicus* var. Gifu ∼ *L. uliginosus ∼ L. corniculatus* (Table 1). No separation with respect to survival was observed between phylogenetically-close and distant genotypes [23], or between model and forage species [21].

**Table 1:** Lethal-dose-fifty (LD50, expressed in days after imbibition) of survival at 300 mM NaCl after a step-wise increase in salt concentration, for each *Lotus* genotype estimated in two independent survival experiments.

Cultivar	LD50Exp_1	LD50Exp_2
*L. japonicus* MG20	66.2	65.0
*L. japonicus* Gifu	41.9	42.0
*L. filicaulis*	54.8	54.6
*L. burttii*	73.8	74.4
*L. corniculatus*	41.0	39.4
*L. glaber*	77.1	76.9
*L. uliginosus*	42.1	41.0

LD50 was calculated fitting the survival data to a Boltzmann sigmoid. Model legume species are shown at the top. Exp  =  experiment.

**Figure 2 pone-0017094-g002:**
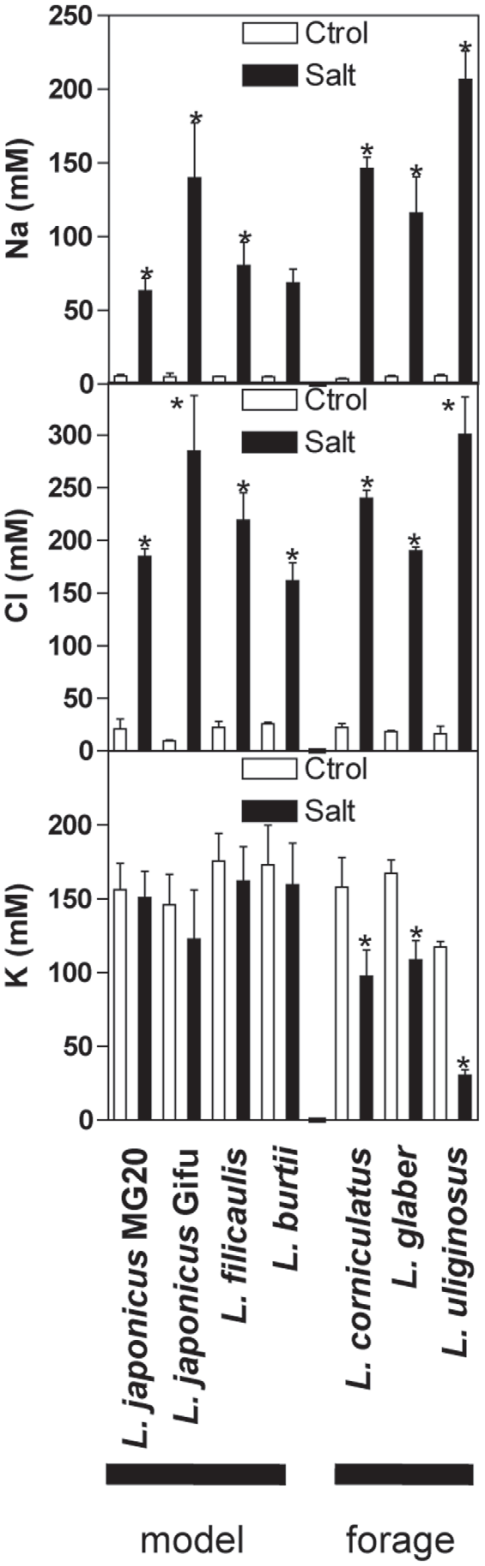
Changes in shoot Na^+^, Cl^−^ and K^+^ in *Lotus* species under salt acclimation. Data represents the mean ± SD of 3 independent experiments. Asterisks indicate a statistically-significant difference (Student *t*-test with adjusted Bonferroni correction, p<0.05) between salt treatment and control.

To facilitate systems comparison under salt acclimation a second treatment regime was applied, which subjected plants to a long-term sub-lethal level of salt (up to 150 mM NaCl, figure 1A, [10]). Three independent experiments were performed, each comprising control and treated plants of each genotype. As expected, shoot biomass decreased under stress (Figure 1C, left panel). Although forage legumes tended to be larger, the relative inhibition of growth was not statistically different between most *Lotus* genotypes (Figure 1C, right panel), and it bore no apparent relationship to the rate of mortality under lethal salt treatment (compare figure 1B, 1C and Table 1). Shoot Na^+^ and Cl^−^ content increased dramatically in all stressed cultivars, exhibiting a negative linear correlation with the LD50 under lethal salinity with a much better correlation coefficient for Cl^−^ levels (Figure 1D and 2). These results are consistent with previous observations that tolerant glycophytes accumulate less salt than sensitive ones [1]–[3], and support the use of LD50 rather than changes in biomass to estimate relative salt tolerance under our experimental conditions. K^+^ concentration changed less in the models than in the forage species in which it decreased 30–70% (Figure 2), and no correlation was found with the LD50.

**Figure 3:**
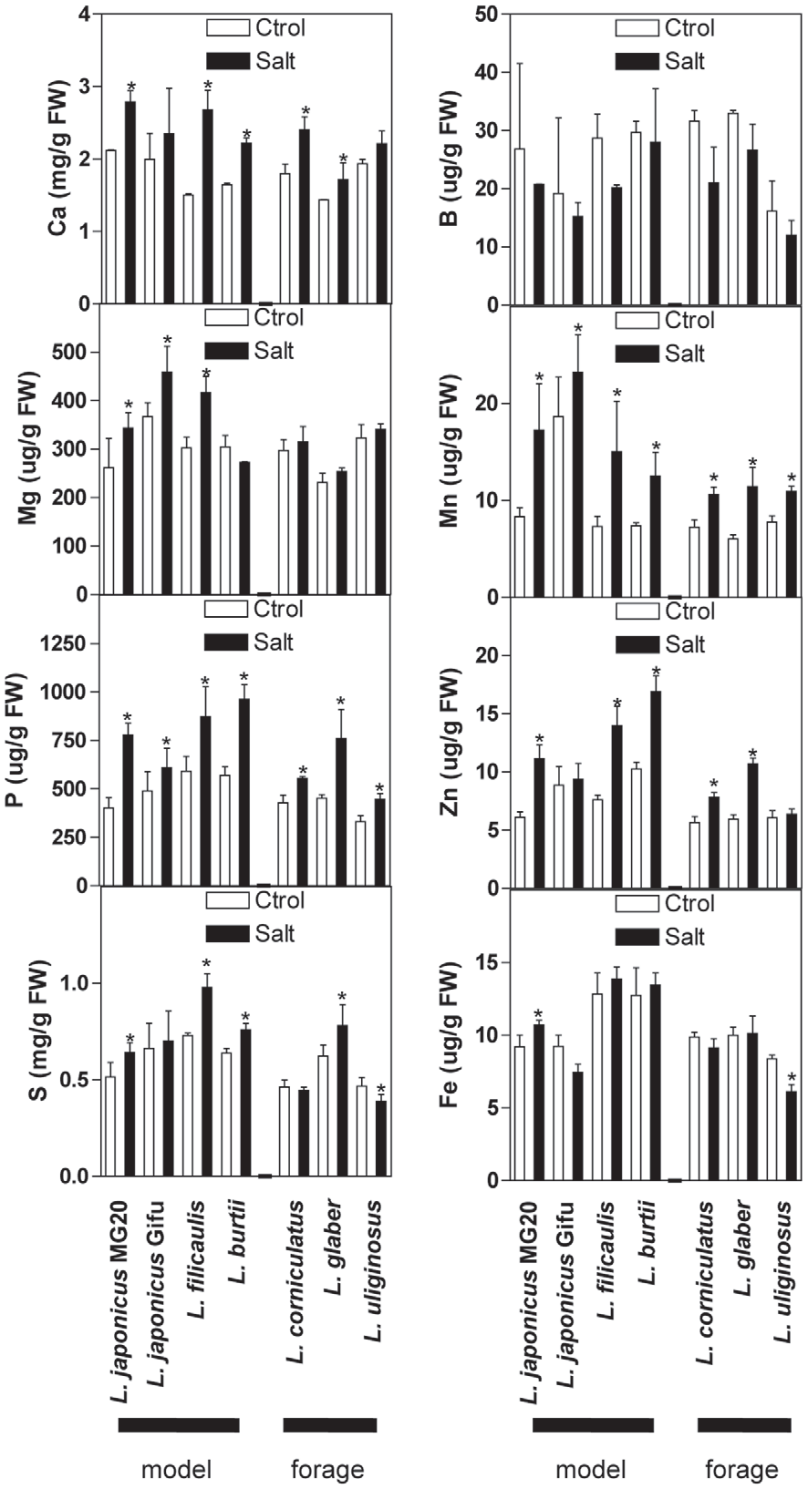
Changes in shoot macro- and micro-nutrients in *Lotus* species under salt acclimation. Data represents the mean ± SD of 3 independent experiments. Asterisks indicate a statistically-significant difference (ANOVA, p<E^−6^) in response to treatment.

Macro- and micro-nutrients were profiled in shoots using ICP-AES, revealing differential salt stress-induced changes in Ca, Mg, Mn, Fe and Zn levels in the different species (Figure 3). No elemental change differentiated model and forage legumes, but the more tolerant cultivars differed from sensitive ones on two ways. First, sulphur increased significantly in tolerant genotypes (*L. glaber*, *L. burtti* and *L. japonicus* var. MG20) but not sensitive ones (*L. japonicus* var. Gifu, *L. uliginosus and L. corniculatus*). Second, although phosphate and zinc content increased significantly in response to NaCl treatment in all or some genotypes, changes were greater in the more tolerant ones (Figure 3).

In summary, physiological and ionomic data revealed complex interactions between NaCl uptake and growth responses, with shoot Cl^−^ levels of stress-acclimated genotypes correlating strongly with rates of mortality in plants exposed to lethal salt-stress doses. No correlation was found between shoot K^+^, Na^+^ or Cl^−^ content and growth inhibition under stress, and nutritional aspects were differentially altered under salinity between more tolerant and sensitive backgrounds. Overall, the data showed a good match between in-breeding model species and out-breeding crop species in their range of tolerance, salt accumulation, variation of nutrient content, and induced growth effects. Thus, the model legume genotypes appear to be valid physiological tools to study and understand salt tolerance mechanisms in the forage species of *Lotus*.

### Gene expression analysis

**Figure 4:**
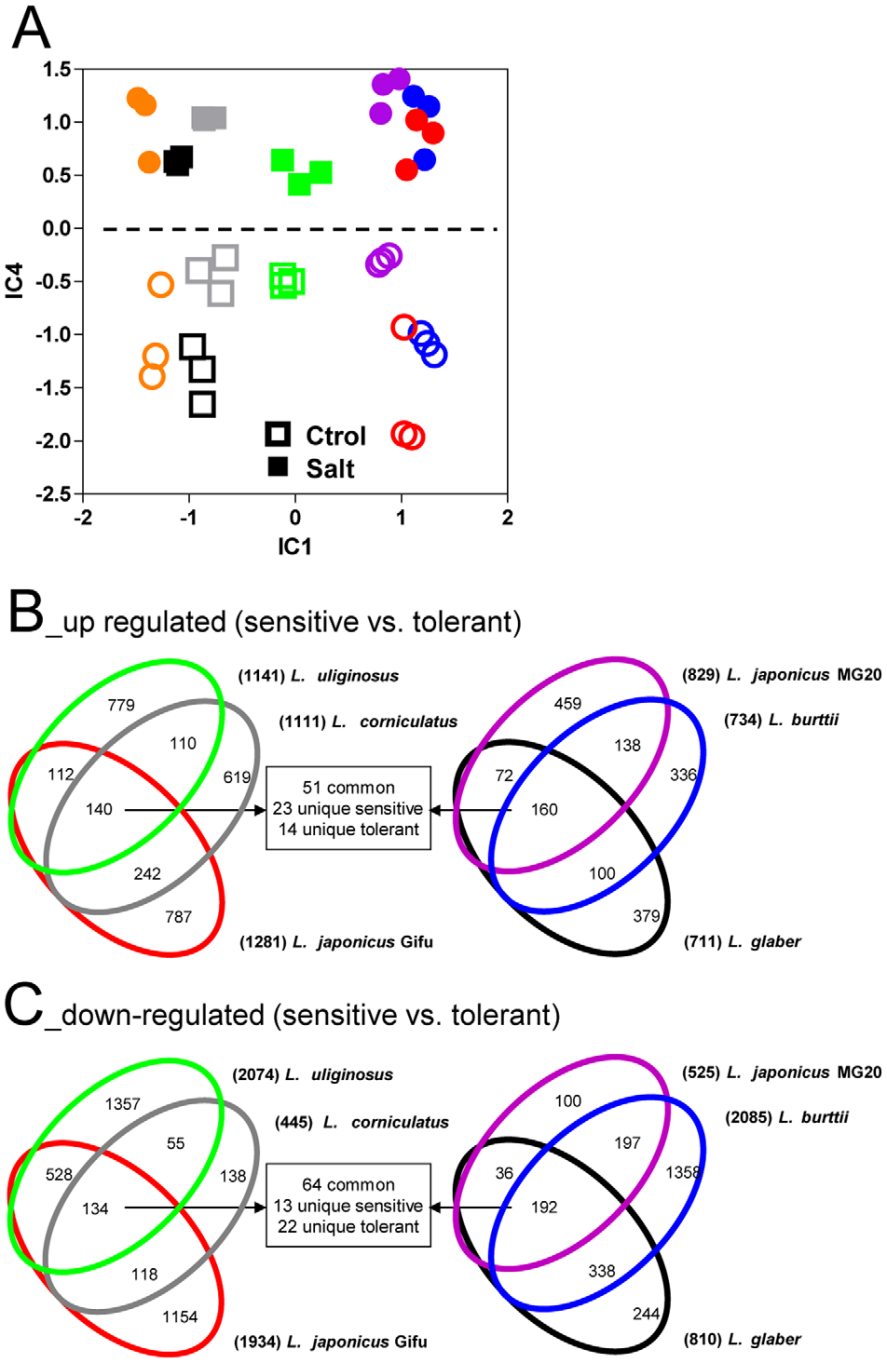
Transcriptional changes in *Lotus* species during salt acclimation. (**A**) Independent component analysis (ICA) of changes in transcript levels. A dashed line separates the symbols that indicate control (empty) and salt acclimation (filled) treatments of the three independent sub-lethal acclimation experiments. (**B** and **C**) Venn diagrams showing the number of salt-induced (B) and repressed (C) genes at FDR<0.05 common to more sensitive and/or tolerant genotypes. *L. filicaulis*, with intermediate tolerance, was excluded (see figure 1 and table S1). Colour code for the different genotypes is as in figure 1.

RNA was isolated from shoots of plants from the sub-lethal salt acclimation experiments, and analyzed using the Affymetrix GeneChip® *Lotus* Genome Array. To avoid problems that might arise from differences in gene/transcript sequences (and, therefore, differences in probe hybridization/signal strength) between species, we ignored data from probe-sets that did not detect transcript in all genotypes and in all three independent experiments, and compared only relative changes in probe-set signal (i.e. ratio Log_2_ Salt/Control for each genotype separately) rather than absolute probe-set signal. Probe-sets that detected transcript in all genotypes, experiments and conditions amounted to 12,137 (Table S1). Non-supervised independent component analysis (ICA) of the whole dataset separated controls from NaCl-treated plants, regardless of the genotype, indicating that at least part of the transcriptional changes were conserved amongst all species (Figure 4A, IC4 represents the shared stress-related variability, while IC1 to IC3 captured genotype-related variability). Data was analyzed by a significance-based test between treated and non-treated plants within each genotype. Of the 12,137 probe-sets called present, 7,776 (64%) detected changes in transcript levels in at least one cultivar upon salt acclimation, but only 92 probe-sets (0.76%) were significantly altered in all seven genotypes (FDR<0.05, Table S1). To facilitate comparisons, the statistically-significant salt-induced or repressed genes were analyzed between the three most sensitive and three most tolerant genotypes, excluding *L. filicaulis* with intermediate tolerance. Remarkably, approximately one-third to one-half of the NaCl-responsive transcripts were specific to a single cultivar (Figure 4B and C). Only 4% of salt-induced genes in the three most-sensitive genotypes were common to all three, while 7% of salt-induced genes in the three most-tolerant genotypes were common to all three. A similar situation was observed for salt-repressed transcripts. On the other hand, many salt-elicited genes common to all sensitive genotypes were also found to be responsive in the more tolerant genotypes (Figure 4B and C, table S2). These shared transcripts included many of unknown function but also genes previously implicated in plant stress such as known *Lotus* stress-responsive genes (LEA protein, phosphatase-2C *LjNPP2C1* and nodulin protein *LjENOD40*, [10]), enzymes of amino acid, polyamine and myo-inositol metabolism (proline oxidase, asparagine synthetase *LjAS1* and histidine decarboxylase, polyamine oxidase *LjPAO4*, S-adenosylmethionine decarboxylase and spermine synthase *LjSPMS*, and myo-inositol phosphate synthase *LjMIPS1*, [10]) and photorespiration (serine-glyoxylate aminotransferase, [24]). We also found tolerant- and sensitive-specific transcripts, most of them of unknown function but some of which have been linked to stress, hormone or nutrient metabolism (Figure 4B and C, table S2). Among others, genes exclusively regulated in tolerant species were involved in stress signalling (phosphatase-2C protein and CBL-interacting protein kinase *LjCIPK6*, [25]), hormone homeostasis (ent-kaurene oxidase *LjGA3*), nitrogen assimilation (cytosolic glutamine synthase *LjGS1*, [26]) and cell wall-related processes such as cellulose synthesis [27]. On the other hand, genes transcriptionally regulated only in salt-sensitive genotypes included also cell wall-related processes, a transcription factor of the DREB sub-family involved in the control of stress responses [28], and the calcineurin B-like protein *LjCBL1* which represent a key node in stress signalling [29]. Homologues of some of the genes described above have been implicated in stress tolerance in other species previously [25], [27]–[31].

In view of the strong link between Cl^−^ concentration and salt tolerance (Figure 1D), we tested for correlations between NaCl-elicited changes in transcript levels and Cl^−^ content under stress for salt-responsive genes shared between all species, and those that were exclusive to tolerant or sensitive genotypes (Figure S1). Remarkably, expression changes of transcripts with shared responses to salinity across species showed little global correlation to Cl^−^ levels. On the other hand, as expected considering the link with survival under lethal salt stress, changes in transcript levels of tolerant- and sensitive- specific genes correlated better with Cl^−^ content.

In summary, the results of transcriptome analysis indicated that a small fraction of the transcriptional responses to salinity were conserved amongst the *Lotus* genotypes. The majority of genes regulated during NaCl acclimation was unique to single genotypes or confined to just a few, and were neither linked to model or forage species nor indicative of the degree of salt tolerance. However, a small sub-set of salt-responsive transcripts were common to all genotypes or represented markers for more tolerant or sensitive genetic backgrounds, and these included key genes involved in stress and metabolism.

### Metabolic phenotype analysis

**Figure 5:**
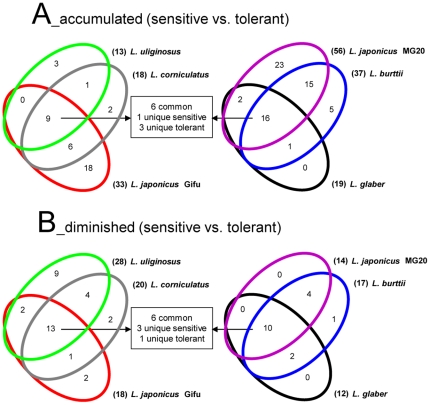
Overview of metabolite profiles of *Lotus* species during salt acclimation. (**A** and **B**) Venn diagrams comparing analytes that were responsive to salt stress in tolerant and sensitive genotypes (Student *t*-test with adjusted Bonferroni correction, p<0.05). *L. filicaulis* was excluded because of its intermediate tolerance (c.f. Figure 1B and table 1). The colour code for the different genotypes is as in figure 1.

**Figure 6 pone-0017094-g006:**
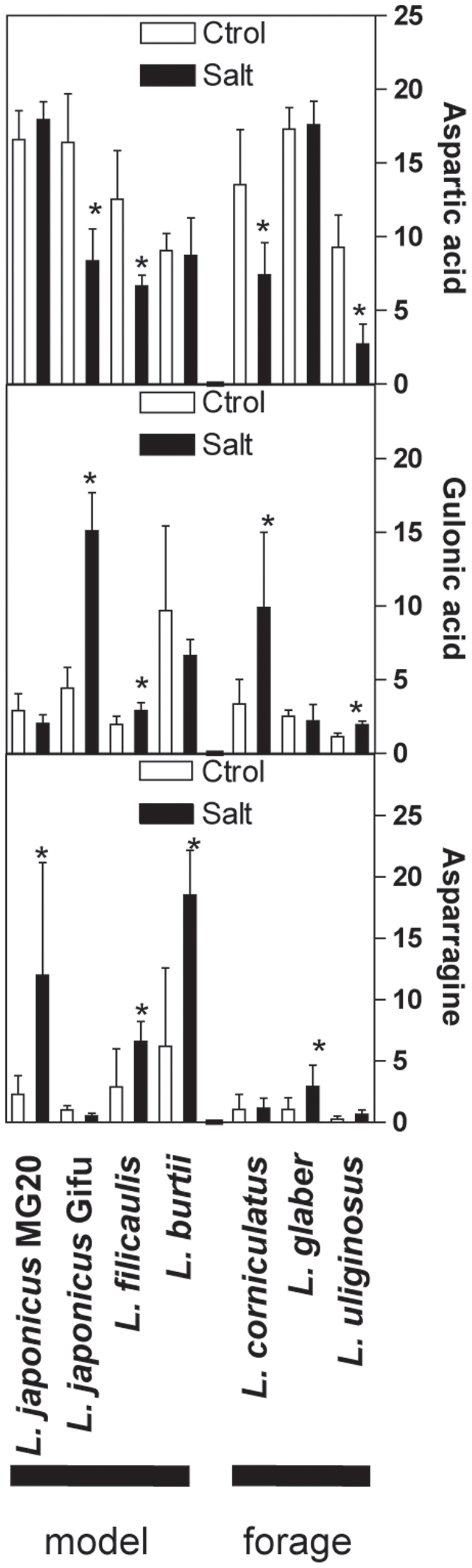
Changes of selected metabolites listed among the markers for sensitive and tolerant genotypes. Data represents the mean ± SD of three independent experiments of the normalized metabolite pools size (i.e. detector signals in arbitrary units normalized to internal standard and sample fresh weight). The asterisks indicate a statistically-significant difference (Student *t*-test with adjusted Bonferroni correction, p<0.05) in the stress-induced change.

The shoot metabolic phenotypes of the *Lotus* species under salt acclimation were determined using non-targeted GC/EI-TOF-MS. A set of 123 analytes, representing both known and unknown compounds, were identified in all genotypes and independent experiments (Table S3). A significance-based analysis was used to determine which analytes responded to salt stress within each genotype (Student *t*-test, p<0.05 adjusted Bonferroni correction). Many NaCl-induced changes in analytes levels were qualitatively similar in most genetic backgrounds, although some metabolic changes were genotype-specific (Figure 5A). Approximately half of the analytes that accumulated under stress were shared between sensitive and tolerant genotypes (Figure 5A, Table S3). These included known *L. japonicus* salt-responsive metabolites such as serine, threonine and ononitol ([10]; Figure S2). On the other hand, levels of organic acids including citric, succinic, malic and threonic acids, declined in most cultivars under salinity, as reported before for model species ([32]; Figure S2). Few salt-induced changes were confined to sensitive genotypes, e.g. increase in gulonic acid and decrease in aspartic acid; or tolerant genotypes, i.e. increase in asparagine (Figure 6). Based on correlation analysis, changes in metabolite levels did not show stable correlative patterns with changes in ion content or biomass under stress, which hampered further integrative analysis. Furthermore, the number of independent microarrays available for each species was insufficient to build robust metabolite-transcript correlations.

**Figure 7:**
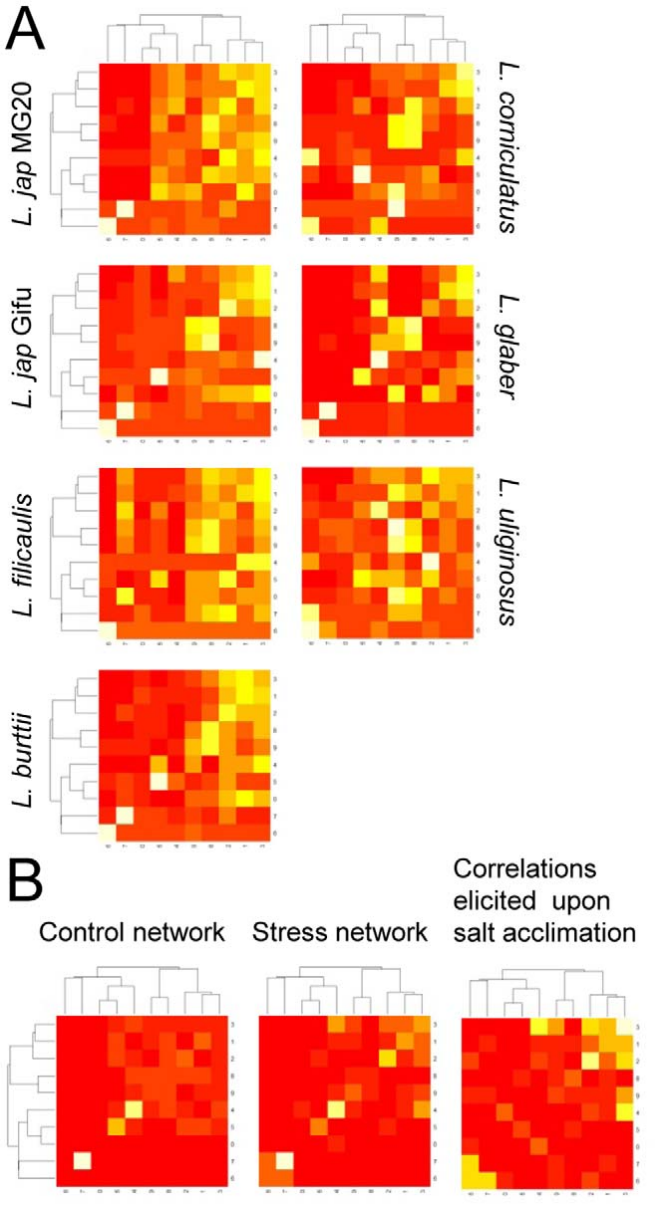
Network analysis of metabolite profiles. (**A** and **B**) Heat-maps representing the statistical connectivity between metabolic network communities: (A) for each genotype, arising from genotype-specific networks, and (B) for the control network, the salt treatment network and for the subtraction of the control and the salt treatment networks (i.e. correlations elicited upon salt stress). Lighter colour (from red to white) represents higher correlation coefficient.

Changes in metabolism were analyzed further, using a metabolic network approach based on pairwise correlations between analyte levels in all genotypes and treatments combined (see methods). Nine communities (modules) of correlated analytes were identified in the resulting network using community structure statistics [33], reflecting conservation of some metabolic modules between genotypes. However, when individual networks were constructed for each cultivar using control and treatment data, clear differences in network architecture were observed, indicating genotype-specific interactions (Figure 7A). Networks for control and salt treatments were also built to identify salt-dependent changes in network architecture that were conserved across genotypes (Figure 7B). Only some of the NaCl-induced communities in the network architecture were shared between genotypes, indicating that some but not all metabolic patterns of response to salt acclimation are conserved between genotypes (compare Figure 7A and B).

In summary, the results indicated that *Lotus* species share many metabolic responses to salinity, and few metabolite markers were found that distinguish tolerant or sensitive genetic backgrounds. On the other hand, several qualitative and quantitative changes under salt-stress, along with some network properties of the metabolome, were unique to individual genotypes.

## Discussion

Previous work has shown that translational genomics can be used to modify traits of agricultural importance, such as pathogen resistance, via a candidate gene approach [34]. But what about complex traits such as tolerance to abiotic stresses, which are determined by the interdependent action of thousands of genes that, in turn, are affected by complex interactions with the environment beyond the stress of interest? We utilised the tools of ionomics, transcriptomics and metabolomics to determine the extent to which plant systems responses to salinity are conserved amongst closely related model and cultivated *Lotus* species [23]. There were no apparent differences between model and forage genotypes of *Lotus* with regard to the range of tolerance/sensitivity, growth inhibition, salt accumulation and nutrient status under salt stress (Figures 1 to [Fig pone-0017094-g002] 3). A strong negative correlation between Cl^−^ levels in the shoot and tolerance to salinity across species (Figure 1D) supported the conclusion that Cl^−^ exclusion from the shoots represents a key physiological mechanism for salt tolerance in legumes, similar to the case of Na^+^ exclusion in other glycophytes [1]–[3], [35]. Taken together, these results indicate that *Lotus* model species respond to salinity in a similar way to crop species and, therefore, are useful systems for identifying physiological processes required for salt tolerance.

Ionomic analysis revealed a differential increase of phosphate, sulphur and zinc in more tolerant genotypes in response to salinity (Figure 3). Presumably, this reflects a differential NaCl-induced imbalance between uptake and translocation to the shoot of these nutrients and plant growth. However, no nutrient correlated with biomass in control or stressed plants. Therefore, the physiological significance of shoot phosphate, sulphur and zinc concentration in *Lotus* shoots during saltinity remains obscure. In fact, these nutrients were observed to decline in other species under salt stress, indicating that the influence of salinity on plant nutrition is variable and dependent on growth conditions, chemical characteristics of the soil and plant genotype [5], [36]–[37].

All *Lotus* genotypes responded to salt stress with massive changes in gene expression. More than 60% of the genes monitored in the shoots of all six species responded to NaCl with significant changes in transcript level in at least one genotype. However, less than 1% responded in all genotypes (Figure 2). Comparisons between salinized root tips of monocot species from different genera indicate that this phenomenon also occurs at the root level [38]. The lack of conservation in the majority of transcriptional responses to salinity reflects at least two things. First, most genes that respond to salinity have little to do with the tolerance of a genus. Second, the architecture of genetic control networks governing transcription is highly complex and variable between genotypes. A similar conclusion was drawn on an expression quantitative trait loci (eQTL) study of recombinant inbred lines of *A. thaliana*, where the majority of eQTL had only small phenotypic effects [39]. The variability in transcriptional responses may also reflect differences in the suite of molecular and cellular mechanisms used to cope with salt accumulation in the shoot, redundancy within multigene families with different genes in the same family fulfilling equivalent roles in different species, and NaCl-responsive genes involved in secondary/pleiotropic responses to stress rather than primary responses required for acclimation. Clearly, simply relying on transcriptional profiling of a single model genotype to identify processes that could be translated to crops would be unwise, in view of the high degree of ‘false positives’ inferred from the above analyses.

On the other hand, by ‘triangulating’ data from multiple species we identified genes that responded in all genotypes, and genes that responded only in salt-tolerant or only in salt-sensitive cultivars. Most NaCl-responsive transcripts that were shared across all genotypes showed little correlation to Cl^−^ levels (Figure S1), indicating that they may be involved in ‘general’ physiological responses such as osmotic stress or growth inhibition [5]. Consistent with this idea, several of these represent key genes of stress-related metabolism, including amino acid, myo-inositol and polyamines biosynthesis, cell wall modification and photorespiration [24], [10], [30]–[31]. Importantly, homologues of at least two of these genes, *AtSPMS* and *AtMIPS1*, contribute to salinity responses in *A. thaliana* [30]–[31]. In contrast to common salt-responsive genes, changes in transcript levels of genes responding only in tolerant or sensitive genotypes showed better correlation with Cl^−^ content (Figure S1), indicating they may be directly or indirectly involved in plant responses to ion accumulation or imbalance. These included homologues of *A. thaliana AtCIPK6* and *AtCBL1*, which are components of signalling pathways required for salt tolerance and members of the CBL-CIPK network controlling nutrient and salt homeostasis [25], [29].

Despite dramatic differences observed at the transcriptome level, about half of the changes in primary metabolism in response to NaCl were shared by all *Lotus* species, although qualitative, quantitative and network analyses revealed several genotype-specific features (Figures 5 to [Fig pone-0017094-g006] 7 and S2). Conserved metabolic changes included increases in the levels of specific amino acids and polyols and decreases in organic acids, most of them recognized as salt-responsive metabolites that may act as compatible solutes or to compensate for ionic imbalance [32]. Changes in gulonic and aspartic acids were confined to sensitive genotypes (Figure 6). The former is an intermediate in the uronic acid pathway that synthesizes the antioxidant ascorbate from myo-inositol [40], and thus an increased gulonic acid content may reflect higher oxidative stress and cellular damage in the sensitive cultivars. If involved in charge balance [32], a decrease in aspartic acid may reflect the higher Cl^−^ content of the sensitive genotypes. On the other hand, asparagine levels increased in the more tolerant genotypes (Figure 6). This metabolite has a central role in the long-distance transport of nitrogen in *Lotus* and it functions at the core of the GS/GOGAT and the ornithine cycles, which are directly or indirectly involved in nitrogen assimilation, proline and polyamine biosynthesis, and ammonium detoxification from photorespiration [26], [41]–[42]. Therefore, asparagine may play a pivotal role in salt tolerance by supporting core nitrogen metabolism. Further research would be needed to test if the manipulation of asparagine metabolism may be useful to improve salt tolerance in legumes.

Although some success has been reported in identifying genes that confer salt tolerance in model plants under controlled conditions using simple molecular biological approaches, little success has been achieved in the field where complex environmental interactions prevail [9], [43]. To complicate things, there are significant differences between plants in how they to cope with saline soils, with salt exclusion, tissue tolerance, and osmotic adjustment playing more or less significant roles in different species [3], [6]. We have shown here that transcriptome and metabolome changes that occur in related model and forage legume species in response to salinity are not highly conserved, which hampers simple translational genomics approaches. A similar conclusion was drawn from *Populus* genotypes subjected to drought, namely that it is not possible to draw simple, generalized conclusions about the stress transcriptome of a genus on the basis of one species [44]. However, a small set of salt-responsive genes were found to be conserved in all *Lotus* genotypes studied here, while salt-responsiveness of other genes was confined to tolerant or sensitive cultivars. It is likely that some of these genes play integral roles in acclimation and tolerance to saline soils; in fact homologues of some have been shown to confer greater salt tolerance in other species. In conclusion, ‘triangulation’ of transcriptomic and metabolomic data from multiple related species/genotypes offers itself as a practical means to eliminate a plethora of false positives in the hunt for genes and processes determining complex traits.

## Materials and Methods

### Plant material, growth conditions and experimental designs

Seeds of *L. japonicus* var. MG20, *L. japonicus* var. Gifu B129, *L. filicaulis, L. burttii* B303, *L. corniculatus* var. San Gabriel, *L. glaber* (*L. tenuis*) var. La Esmeralda and *L. uliginosus* var. LE G27 were obtained from the LOTASSA consortium (www.lotassa.org). Seeds were germinated in half-strength BD solution [45] agar plates plus 2 mM KNO_3_ and 2 mM NH_4_NO_3_. Four days after imbibition, seedlings were transplanted to soil (Einheit, type null) using 10 cm pots irrigated with the above solution and grown in greenhouse under 16/8 hours day/night, 23±2°C and 55–65% RH. Salt stress treatment started 8 days post-imbibition and the salt content in the nutrient solution was increased in steps of 4 days till reaching the desired concentration (300 or 150 mM NaCl) (Figure 1A). Fresh nutrient solution was prepared every 4 days. Survival experiments were performed at lethal salt stress doses (300 mM NaCl) and were repeated twice, measuring the rate of mortality which was scored when the whole plant or all leafs were wilted or chlorotic. For salinity acclimation, 3 successive independent sub-lethal salt stress experiments were performed (150 mM NaCl), each consisting of 14 sample sets comprising control and salt treatments for each of the genotypes. Each set had 6 independent biological replicate pools of 5 plants. Total time of culture was 32 days and whole shoots, excluding cotyledons, were harvested *in situ* into liquid nitrogen in the middle of the light period. At harvest all plants were in the vegetative stage, with roots never showing nodules. The exposition to an identical salt stress dose between the genotypes was confirmed measuring the soil conductivity [10]. Biomass was estimated by mean fresh weight of the pooled shoots.

### Profiling analysis

Transcriptomic, metabolomic and ionomic profiling were performed as described previously [10]. For the transcriptiome analysis, sample tissue of all the biological replicates of each genotype were pooled to obtain 14 representative RNA samples in each independent experiment. The resulting 42 RNA samples were labelled and hybridized to the Genechip® Lotus1a520343 (Affymetrix). Element and metabolites and content were determined in each biological replicate using ICP-AES and GC/EI-TOF-MS, respectively. For ionomic profiling, 100 mg plant material was digested with 2 ml HNO_3_ at 140°C until complete digestion. 100 µl of a 100 g/L LiCl solution was added as a carrier and the final volume adjusted with ultra pure water to 10 ml. Element concentrations were determined with inductively coupled plasma-atomic emission spectrometry (ICP-AES) using an IRIS Advantage Duo ER/S (Thermo Fisher). Elemental quantification was validated using IC-CTA-VTL2 Virginia tobacco leaves as a certified reference material. Chloride was profiled using ion chromatography with a Dionex ICS-2000 system (Dionex). For metabolomic profiling, 60 mg of frozen plant tissue was extracted with methanol/chloroform, and the polar fraction was prepared by liquid partitioning into water and derivatized [46]. Gas chromatography coupled to electron impact ionization-time of flight-mass spectrometry (GC/EI-TOF-MS) was performed using an Agilent 6890N24 gas chromatograph with split or splitless injection mounted to a Pegasus III time-of-flight mass spectrometer (LECO) [47]. Metabolite-features were quantified after mass spectral deconvolution (ChromaTOF software 1.00, Pegasus driver 1.61, LECO), and their chemical identification was manually assessed using the NIST05 software (http://www.nist.gov/srd/mslist.html) and the mass spectral and retention time index collection of the Golm Metabolome Database [48].

### Statistics, data and network analyses

Statistical differences between control and treatments in element content were assessed with two-way-ANOVA using “treatment” and “independent experiment” as factors at stringent statistical threshold (p<E^−6^), with the TIGR multiple experiment viewer software (TMEV_3.1). Microarray data were normalized by the GC-RMA algorithm using the bioconductor package of R software. Differential expression was tested for the probesets called present in all experiments, species and treatments (12,137 probesets, according to the present/absent MAS5 algorithm) correcting for multiple testing across all genes using the linear step-up false discovery rate (FDR). Microarray data is MIAME compliant and the 42 hybridizations were deposited at Array-Express (www.ebi.ac.uk/arrayexpress, accession number E-MEXP-2344). Independent component analysis (ICA) was used as non-supervised clustering algorithm, through the MetaGeneAlyse webpage (http://metagenealyse.mpimp-golm.mpg.de). Correlations across experiments and genotypes between the salt-elicited fold change in gene expression (Log_2_ Salt/Control) and Cl^−^ content under stress were assessed using the Pearson correlation. Metabolomic profiles were analyzed with the TagFinder software [49] and filtered for those metabolic-features represented by 3 or more inter-correlated mass fragments within each independent experiment [50]. The validity of this analytical approach to quantify metabolites in plant tissues have been previously demonstrated [51]. Resulting data was normalized to internal standard and fresh weight, and each metabolic-feature was normalized to the median within each experiment and genotype, and log_10_ transformed prior to statistical analysis. Statistical differences were assessed with Student *t*-test using TMEV_3.1 (p<0.05 applying the adjusted Bonferroni correction). Network analysis and operations were performed using R and Pajek softwares. A stable metabolic backbone network was reconstructed in two steps [52]. The first step recognized highly correlated metabolites through the construction of a “union network” based on a Spearmann rank-order analyte-analyte correlation in each cultivar for the 123 identified analytes, which was transformed into binary matrices according to a p<E^−4^ threshold (applying Bonferroni correction) and considered further if it was significant in at least one cultivar. In the second step, a homogeneity test of the distributions of the correlations coefficients (Z-score transformed) was performed for all analyte-analyte correlations of the union network. Only those with a significant threshold (p<E^−6^) in a Chi-square test with were considered stable. As a consequence, the reconstruction of a backbone network represents statistically stable correlated analytes between species. Particular networks for each genetic background or for control and treatment conditions were reconstructed based on this stable backbone. Tightly connected clusters of metabolites within the stable network (communities) were detected using Newman's algorithm for modularity, establishing an arbitrary number when modularity gain reached a plateau [33].

## Supporting Information

Table S1 Transcriptomic profile data.
(XLS)

Table S2 Salt-elicited genes which were shared between more sensitive and tolerant genotypes, or were specific for more tolerant or sensitive backgrounds.
(XLS)

Table S3 Manually identified analytes present in all three independent non-lethal salt stress acclimation experiments.
(XLS)

Figure S1 Correlation (Pearson coefficients) across experiments and genotypes between changes in gene expression (Log2 Salt/Control) and Cl^−^ content under stress.
(TIF)

Figure S2 Example of metabolites that responded to sub-lethal salt stress in the different *Lotus* species.
(TIF)

## References

[1] Tester M, Davenport R (2003). Na^+^ tolerance and Na^+^ transport in plants.. Ann Bot.

[2] Munns R (2005). Genes and salt tolerance: bringing them together,. New Phytol.

[3] Munns R, Tester M (2008). Mechanisms of salinity tolerance.. Ann Rev Plant Biol.

[4] Achard P, Cheng H, De Grauwe L, Decat J, Schoutteten H (2006). Integration of plant responses to environmentally activated phytohormonal signals.. Science.

[5] Sanchez DH, Szymanski J, Erban A, Udvardi MK, Kopka J (2010). Mining for robust transcriptional and metabolic responses to long-term salt stress: a case study on the model legume *Lotus japonicus*.. Plant Cell Environm.

[6] Moller IS, Tester M (2007). Salinity tolerance of Arabidopsis: a good model for cereals?. Trends Plant Sci.

[7] Monforte AJ, Asins MJ, Carbonell EA (1997). Salt tolerance in *Lycopersicon* species. VI. Genotype-by-salinity interaction in quantitative trait loci detection: constitutive and response QTLs.. Theor Appl Gen.

[8] Foolad MR (2004). Recent advances in genetics of salt tolerance in tomato.. Plant Cell Tiss Org Culture.

[9] Cuartero J, Bolarin MC, Asins MJ, Moreno V (2006). Increasing salt tolerance in the tomato.. J Exp Bot.

[10] Sanchez DH, Lippold F, Redestig H, Hannah M, Erban A (2008). Integrative functional genomics of salt acclimatization in the model legume Lotus japonicus.. Plant J.

[11] Graham PH, Vance CP (2003). Legumes: importance and constraints to greater use. Plant Physiol.

[12] Young ND, Udvardi MK (2009). Translating *Medicago truncatula* genomics to crop legumes.. Curr Opin Plant Biol.

[13] Cannon SB, Sterck L, Rombauts S, Sato S, Cheung F (2006). Legume genome evolution viewed through the Medicago truncatula and Lotus japonicus genomes.. Proc Nat Acad Sci.

[14] Perry JA, Wang TL, Welham TJ, Gardner S, Pike JM (2003). A TILLING reverse genetics tools and a web-accessible collection of mutants of the legume *Lotus japonicus.*. Plant Physiol.

[15] Udvardi MK, Tabata S, Parniske M, Stougaard J (2005). *Lotus japonicus*: legume research in the fast lane.. Trends Plant Sci.

[16] Gondo T, Sato S, Okumura K, Tabata S, Akashi R (2007). Quantitative trait locus analysis of multiple agronomic traits in the model legume *Lotus japonicus*,. Genome.

[17] Sato S, Nakamura N, Kaneko T, Asamizu E, Kato T (2008). Genome structure of the legume, *Lotus japonicus.*. DNA Research.

[18] Høgslund N, Radutoiu S, Krusell L, Voroshilova V, X Hannah MA (2009). Dissection of symbiosis and organ development by integrated transcriptome analysis of *Lotus japonicus* mutant and wild-type plants.. PLoS One.

[19] Diaz P, Betti M, Sanchez DH, Udvardi MK, Monza J (2010). Deficiency in plastidic glutamine synthetase alters proline metabolism and trasncriptomic response in *Lotus japonicus* under drought stress.. New Phytol.

[20] Kawaguchi M, Pedrosa-Harand A, Yano K, Hayashi M, Murooka Y (2005). *Lotus burttii* takes a position of the third corner in the *Lotus* molecular genetics triangle.. DNA Research.

[21] Diaz P, Borsani O, Monza J, Marquez A (2005). Lotus-related species and their agronomic importance..

[22] Sanchez DH, Cuevas JC, Chiesa MA, Ruiz OA (2005). Free spermidine and spermine content in *Lotus glaber* under long-term salt stress.. Plant Sci.

[23] Degtjareva GV, Kramina DD, Sokoloff DD, Samigullin TH, Valiejo-Roman CM (2006). Phylogeny of the genus *Lotus* (Leguminoseae, Loteae): evidence from nrITS sequences and morphology.. Can J Bot.

[24] Noctor G, Veljovic-Jovanovic S, Driscoll S, Novitskaya L, Foyer CH (2002). Drought and oxidative load in the leaves of C_3_ plants: a predominant role for photorespiration?. Ann Bot.

[25] Tripathi V, Parasuraman B, Laxmi A, Chattopadhyay D (2009). CIPK6, a CBL-interacting protein kinase is required for development and salt tolerance in plants.. Plant J.

[26] Marquez AJ, Betti M, Garcia-Calderon M, Pal'ove-Balang P, Diaz P (2005). Nitrate assimilation in *Lotus japonicus.*. J Exp Bot.

[27] Chen ZZ, Hong XH, Zhang HR, Wang YQ, Li X (2005). Disruption of the cellulose synthase gene, AtCesA8/IRX1, enhances drought and osmotic stress tolerance in Arabidopsis.. Plant J.

[28] Nakano T, Suzuki K, Fujimura T, Shinshi H (2006). Genome-wide analysis of ERF gene family in Arabidopsis and Rice.. Plant Physiol.

[29] Albrecht V, Weinl S, Blazevic D, D'Angelo C, Batistic O (2003). The calcium sensor CBL1 integrates plant responses to abiotic stresses.. Plant J.

[30] Yamaguchi K, Takahashi Y, Berberich T, Imai A, Miyazaki A (2006). The polyamine spermine protects against high salt stress in *Arabidopsis thaliana*.. FEBS Lett.

[31] Donahue JL, Alford SR, Torabinejad J, Kerwin RE, Nourbakhsh A (2010). The *Arabidopsis thaliana* myo-inositol 1-phosphate synthase1 gene is required for myo-inositol synthesis and suppression of cell death.. Plant Cell.

[32] Sanchez DH, Siahpoosh MR, Roessner U, Udvardi MK, Kopka J (2008). Plant metabolomics reveals conserved and divergent metabolic responses to salinity.. Physiol Plant.

[33] Newman MEJ (2006). Modularity and community structure in networks.. Proc Nat Acad Sci.

[34] Salentijn EMJ, Pereira A, Angenent GC, Van der Linden GC, Krens F (2007). Plant translational genomics: from model species to crops.. Mol Breeding.

[35] Teakle NL, Tyerman SD (2010). Mechanisms of Cl^−^ transport contributing to salt tolerance.. Plant Cell Envirom.

[36] Marschner H (1995). In *Mineral nutrition of higher plants* 2^nd^ edn (Academic Press Limited, London)..

[37] Grattan SR, Grieve CM, Pessarakli M (1999). Mineral nutrient acquisition and response by plants grown in saline environments..

[38] Walia H, Wilson C, Ismail AM, Close TJ, Cui X (2009). Comparing genomic expression patterns across plant species reveals highly diverged transcriptional dynamics in response to salt stress.. BMC Genomics.

[39] West MAL, Kim K, Kliebenstein DJ, van Leeuwen H, Michelmore RW (2007). Global eQTL mapping reveals the complex genetic architecture of transcript-level variation in Arabidopsis.. Genetics.

[40] Ishikawa T, Dowdle J, Smirnoff N (2006). Progress in manipulating ascorbic acid biosynthesis and accumulation in plants.. Physiol Plant.

[41] Sieciechowicz KA, Joy KW, Ireland RJ (1988). The metabolism of asparagine in plants.. Phytochemistry.

[42] Waterhouse RN, Smyth AJ, Massonneau A, Prosser IM, Clarkson DT (1996). Molecular cloning and characterization of asparagine synthetase from *Lotus japonicus*: dynamics of asparagine synthesis in N-sufficient conditions.. Plant Mol Biol.

[43] Flowers TJ (2004). Improving crop salt tolerance.. J Exp Bot.

[44] Wilkings O, Waldron L, Nahal H, Provart NJ, Campbell MM (2009). Genotype and time of the day shape the *Populus* drought response.. Plant J.

[45] Broughton WJ, Dilworth MJ (1971). Control of leghaemoglobin synthesis in snake beans.. Biochem J.

[46] Desbrosses GG, Kopka J, Udvardi MK (2005). *Lotus japonicus* metabolic profiling. Development of gas chromatography-mass spectrometry resources for the study of plant-microbe interactions.. Plant Physiol.

[47] Wagner C, Sefkow M, Kopka J (2003). Construction and application of a mass spectral and retention time index database generated from plant GC/EI-TOF-MS metabolite profiles.. Phytochemistry.

[48] Kopka J, Schauer N, Krueger S, Birkemeyer C, Usadel B (2005). GMD@CSB.DB: the Golm Metabolome Database.. Bioinformatics.

[49] Luedemann A, Strassburg K, Erban A, Kopka J (2008). TagFinder for the quantitative analysis of gas chromatography-mass spectrometry (GC-MS) based metabolite profiling experiments.. Bioinformatics.

[50] Sanchez DH, Redestig H, Krämer U, Udvardi MK, Kopka J (2008c). Metabolome-ionome-biomass interactions: what can we learn about salt stress by multiparallel phenotyping?. Plant Sig Behav.

[51] Allwood JW, Erban A, de Koning S, Dunn WB, Luedemann A (2009). Inter-laboratory reproducibility of fast gas chromatography-electron impact-time of flight mass spectrometry (GC-EI-TOF/MS) based plant metabolomics.. Metabolomics.

[52] Szymanski J, Jozefczuk S, Nikoloski Z, Selbig J, Nikiforova V (2009). Stability of metabolic correlations under changing environmental conditions in *Escherichia coli* - a systems approach.. PLoS One.

