# Supplementary figures and images for: Comparative Functional Genomics of Salt Stress in Related Model and Cultivated Plants Identifies and Overcomes Limitations to Translational Genomics
Source: PLoS One. 2011 Feb 14;6(2):e17094. doi: 10.1371/journal.pone.0017094 (PMC3038935; doi:10.1371/journal.pone.0017094)

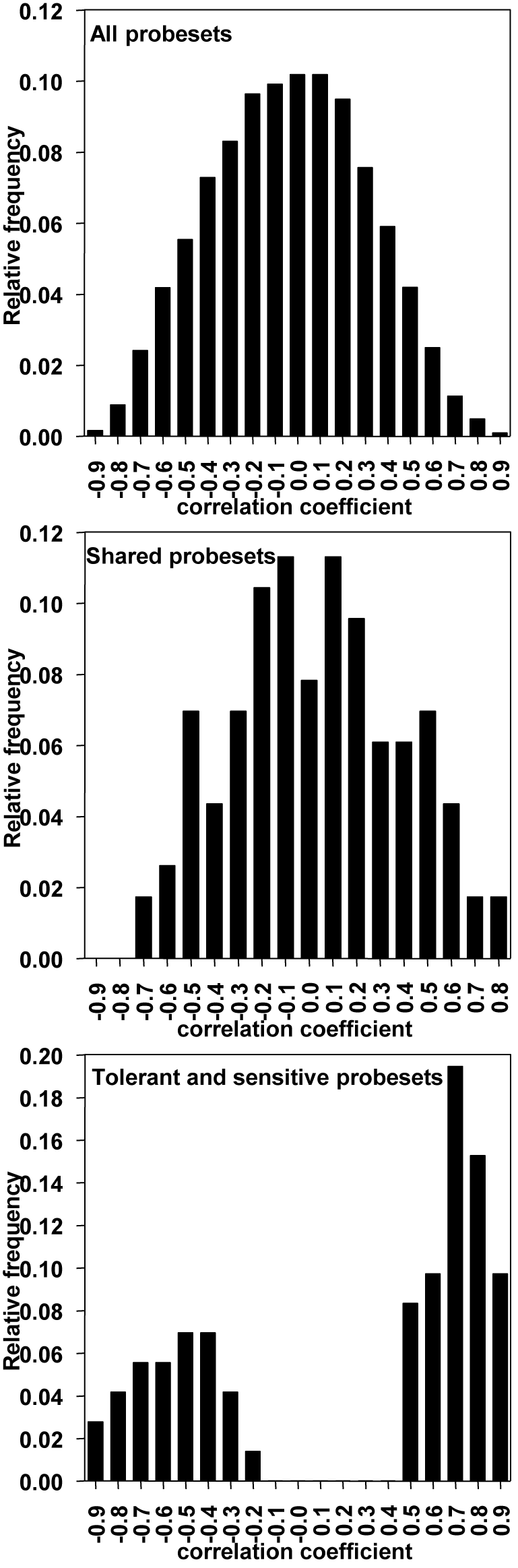

Supplement: Figure S1 — Correlation (Pearson coefficients) across experiments and genotypes between changes in gene expression (Log2 Salt/Control) and Cl− content under stress. (TIF) [file pone.0017094.s004.tif]

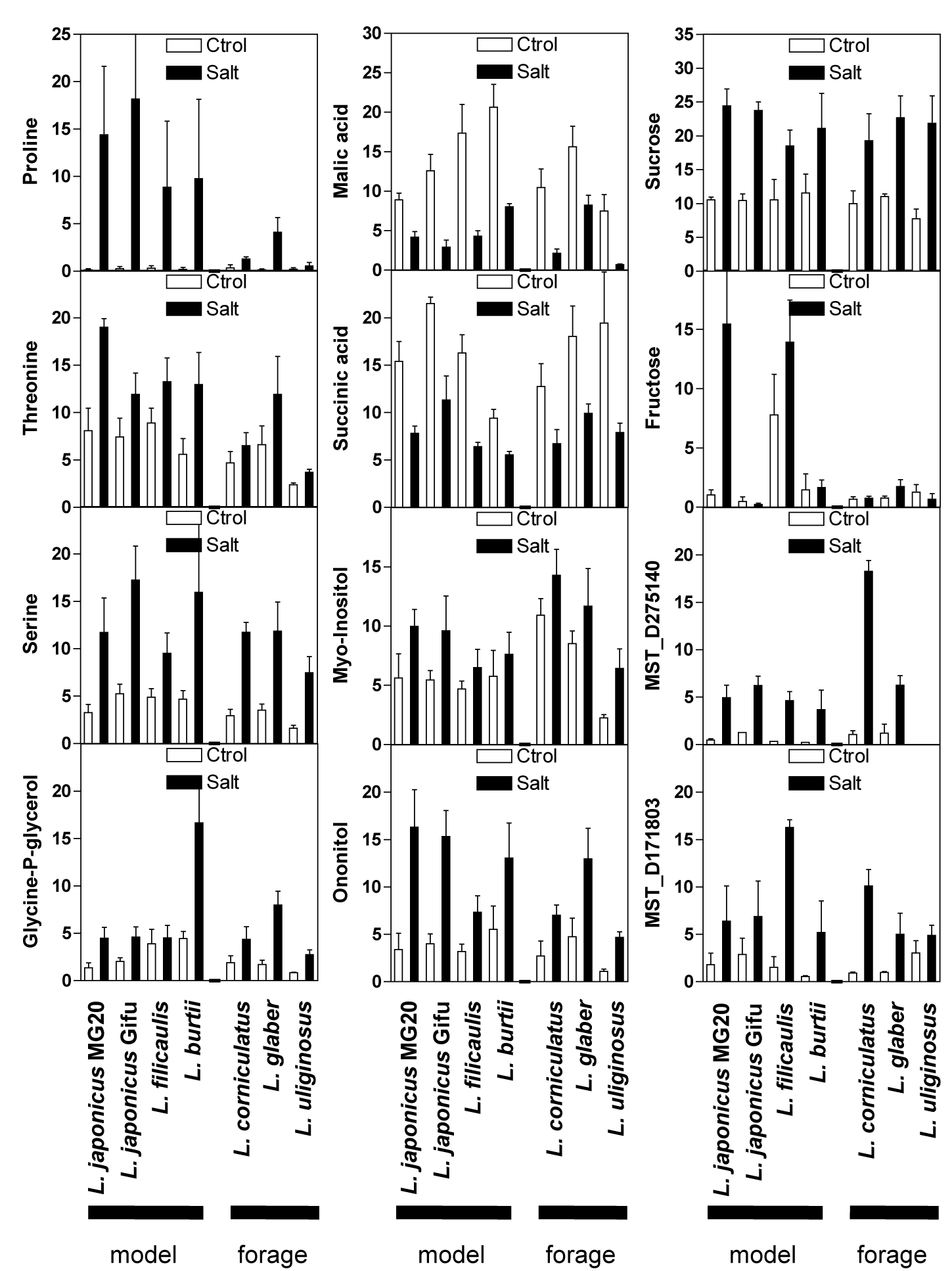

Supplement: Figure S2 — Example of metabolites that responded to sub-lethal salt stress in the different Lotus species. (TIF) [file pone.0017094.s005.tif]
